# The relationship between glucose and the liver-alpha cell axis – A systematic review

**DOI:** 10.3389/fendo.2022.1061682

**Published:** 2023-01-05

**Authors:** Thomas Pixner, Nathalie Stummer, Anna Maria Schneider, Andreas Lukas, Karin Gramlinger, Valérie Julian, David Thivel, Katharina Mörwald, Harald Mangge, Christopher Dalus, Elmar Aigner, Dieter Furthner, Daniel Weghuber, Katharina Maruszczak

**Affiliations:** ^1^ Department of Pediatric and Adolescent Medicine, Salzkammergutklinikum Voecklabruck, Voecklabruck, Austria; ^2^ Obesity Research Unit, Paracelsus Medical University, Salzburg, Austria; ^3^ Department of Pediatrics, Paracelsus Medical University, Salzburg, Austria; ^4^ Department of Sport Medicine and Functional Explorations, Diet and Musculoskeletal Health Team, Human Nutrition Research Center (CRNH), INRA, University Hospital of Clermont-Ferrand, University of Clermont Auvergne, Clermont-Ferrand, France; ^5^ Laboratory of Metabolic Adaptations to Exercise under Physiological and Pathological Conditions (AME2P), University of Clermont Auvergne, Clermont-Ferrand, France; ^6^ Clinical Institute of Medical and Chemical Laboratory Diagnostics, Medical University of Graz, Graz, Austria; ^7^ First Department of Medicine, Paracelsus Medical University, Salzburg, Austria

**Keywords:** liver-alpha cell axis, glucagon, glucose, liver, OGTT, pediatric

## Abstract

Until recently, glucagon was considered a mere antagonist to insulin, protecting the body from hypoglycemia. This notion changed with the discovery of the liver-alpha cell axis (LACA) as a feedback loop. The LACA describes how glucagon secretion and pancreatic alpha cell proliferation are stimulated by circulating amino acids. Glucagon in turn leads to an upregulation of amino acid metabolism and ureagenesis in the liver. Several increasingly common diseases (e.g., non-alcoholic fatty liver disease, type 2 diabetes, obesity) disrupt this feedback loop. It is important for clinicians and researchers alike to understand the liver-alpha cell axis and the metabolic sequelae of these diseases. While most of previous studies have focused on fasting concentrations of glucagon and amino acids, there is limited knowledge of their dynamics after glucose administration. The authors of this systematic review applied PRISMA guidelines and conducted PubMed searches to provide results of 8078 articles (screened and if relevant, studied in full). This systematic review aims to provide better insight into the LACA and its mediators (amino acids and glucagon), focusing on the relationship between glucose and the LACA in adult and pediatric subjects.

## 1 Introduction

The prevalence of obesity, non-alcoholic fatty liver disease (NAFLD) and type 2 diabetes mellitus (T2DM) are steadily rising in adults as well as in pediatric populations worldwide (1). NAFLD is considered as the most common chronic liver disease across all age groups (2–6).

Research on the liver-alpha cell axis (LACA) has shed new light on the pathophysiology of NAFLD and proposed glucagon and amino acids as pivotal drivers (7–9). Glucagon is predominantly known for its counter-regulatory mechanism of insulin and the ability to coordinate blood glucose homeostasis. While hypoglycemia stimulates the secretion of glucagon from pancreatic alpha cells to promote gluconeogenesis and glycogenolysis in the liver, glucagon’s role is more diverse (10). It has been demonstrated that glucagon administration leads to a reduction in plasma amino acids (AAs) and to increased AA-uptake *via* the liver and consequently to conversion into gluconeogenic precursors both in animals and humans (11, 12). In contrast, evidence that the liver and the pancreatic alpha cells are linked in a feedback-cycle is recent (8, 13). In 2015, Solloway et al. demonstrated that inhibiting the glucagon-receptor increases AA concentrations, reduces AA turnover, and promotes pancreatic alpha cell proliferation (14–16). Further research by Kim et al. in 2017 involving antibody-mediated blockage of the glucagon-receptor led to the discovery of slc38a5. This gene is upregulated in the liver and pancreas during the blockade and encodes an AA transporter favoring neutral amino acids (e.g., glutamine). Slc38a5-deficient mice showed diminished alpha cell proliferation in response to glucagon receptor blockade (17). Furthermore, mTORC1 regulates the slc38a5 expression, and inhibits mTORC1 by rapamycin blocked AA-induced alpha cell proliferation (7, 18, 19). Holst, Wewer Albrechtsen, and Pedersen postulated a feedback regulation mechanism that links the liver and the pancreatic alpha cell known as LACA (8, 9).

**Figure 1:**
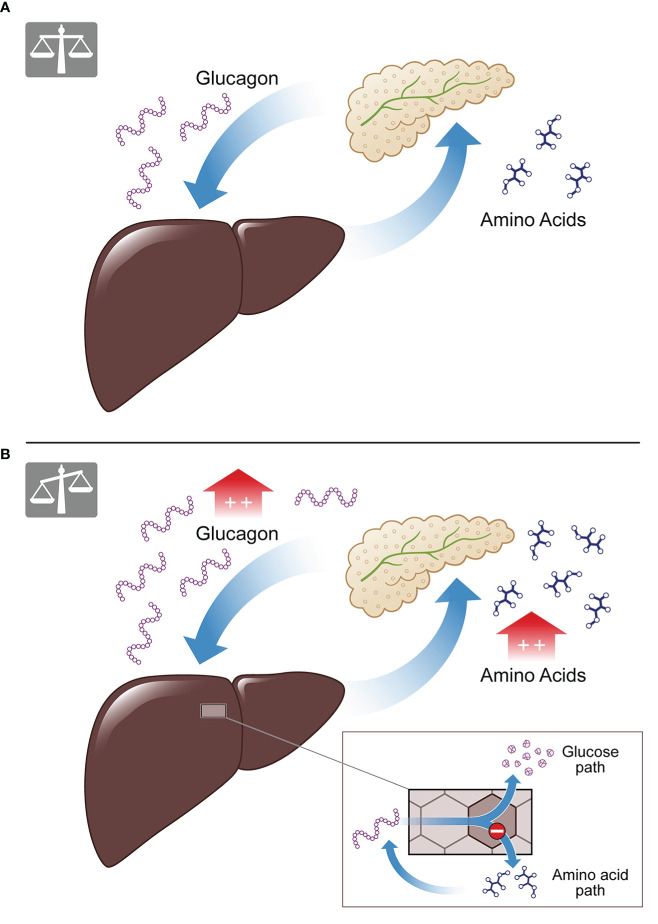
The liver-alpha cell axis. **(A)** Under physiological conditions amino acids lead to glucagon secretion from the pancreatic alpha cell, causing amino acid degradation and ureagenesis in the liver. The result is a balanced feedback cycle, termed the liver-alpha cell axis. **(B)** In conditions of metabolical impairment (e.g., NAFLD, T2D) the cycle is malfunctioning. Amino acids cannot be properly degraded as the amino acid pathway is disrupted, while the glucose pathway of glucagon is functioning (see insert). This results in hyperaminoacidemia and hyperglucagonemia.

The LACA describes, under physiological conditions, how a rise of plasmatic AAs induces glucagon secretion from the pancreatic alpha cells. Postprandial glucagon, in turn, controls hepatic AA metabolism and induces ureagenesis, resulting in decreasing levels of plasmatic AAs. When levels of plasmatic AAs return to their normal range, so does glucagon (see **Figure 1A**) (9, 20, 21). It is of special interest that the LACA seems to be disturbed in obesity and associated conditions. Besides genetic defects and pharmacological interventions to inhibit glucagon signaling, hepatic disorders (e.g., NAFLD and NASH (9, 22, 23)) and certain metabolic conditions (e.g., obesity (24, 25), T2DM (22)) have been associated with impaired glucagon signaling and hence disrupted LACA (26, 27). Under such conditions, the weakened effect of glucagon on AA turnover results in reduced ureagenesis and hyperaminoacidemia. Elevated levels of plasmatic AAs, in turn, lead to hyperglucagonemia and proliferation of pancreatic alpha cells. This process creates a vicious cycle of metabolic imbalance (see **Figure 1B**) (10, 11, 14, 17, 22, 23, 28).

While amino acids are drivers of the LACA, study results on specific AAs remain controversial. Most AAs stimulate glucagon and insulin secretion; however, some are more potent (29–34). Glucagon is the second mediator of the LACA, and its secretion is a complex process in itself and regulated by multiple interactions. These include changes in blood glucose, vegetative (sympathetic and parasympathetic) and other neural signals, amino acids, fatty acids, endocrine and paracrine functions (18, 35–39). A disturbed LACA seems to mark glucagon resistance in the hormone AA-pathway (15, 22), resulting in elevated plasmatic levels of amino acids, while the glucose pathway appears to remain intact (10, 13, 14, 17, 20, 21, 40–42). Hyperglucagonemia results in increased hepatic glucose production (43) *via* maintained glycogenolysis (21), and increased activity of gluconeogenesis’ key enzymes (9). In obesity, NAFLD, and T2DM, fasting hyperglucagonemia is common and stimulates hepatic glucose production, predisposing to elevated blood glucose levels (44–47). The diabetogenic role of glucagon is widely accepted and confirmed in patients with type 2 diabetes (48–50). If not counterbalanced, hyperglycemia may result in insulin resistance, worsening of NAFLD and the development of T2DM and metabolic syndrome. Hence, understanding how metabolic risk factors such as (increased) glucose intake may affect the LACA is essential. Additionally, AA and glucagon levels during oral glucose tolerance test (OGTT) may hold predictive/diagnostic information on metabolically associated diseases.

Besides providing an overview of the LACA, the aim of this systematic review is to summarize the relationship between glucose and the mediators of the LACA (i.e., glucagon and AAs) under physiological conditions and in conditions of metabolical impairment. This review is structured to report data of the mediators` interactions with glucose and each other as well as their dynamics during experimental glucose administration including results from the pediatric population.

## 2 Methods

### 2.1 Design

This systematic review was conducted and written following the Preferred Reporting Items for Systematic Reviews and Meta-Analysis (PRISMA) guidelines (49). Inclusion criteria were quantitative studies, peer reviewed-papers, basic research, and clinical research. We excluded non-English publications and studies with a focus other than human medicine (e.g., veterinary medicine).

### 2.2 Search strategy

Three authors independently searched the PubMed database and cooperated in screening the results. The purpose of the search was to identify data on LACA and its relationship with glucose. As glucagon and amino acids are the LACA’s main mediators, these were of central interest concering glucose-interaction. The first search was conducted on December 13th and 14th, 2021. The first search included the terms “liver AND alpha cell AND axis,” “liver-alpha cell,” “Liver-islet axis,” “Glucagon AND liver,” and “Glucose and amino acids” without any parameters (e.g., age) in all articles up to December 1st, 2021. A second search on December 22nd, 2021, included “OGTT Amino acids” (articles from 1.1.2016-12.1.2021) and “OGTT Glucagon” (articles from 1.1.2016-12.1.2021) without additional filters. The rationale for the search period was the keystone study on glucagon-receptor blockade and alpha cell hyperplasia by Solloway et al. in July 2015, the report of hyperglucagonemia in NAFLD in 2016 and postulation of LACA in 2018 (14, 15, 20). As part of the second search (“OGTT Amino acids” and “OGTT Glucagon”) studies on pediatric cohorts (0-18 years) published between 1.1.2000-12.31.2021 were searched. The search period for pediatric results was extended until the beginning of the year 2000 due to the lack of pediatric studies and difficulties with glucagon measurements in older studies (51, 52). For the second search inclusion criteria were the same as in the first search attempt. However, exclusion criteria were extended to pregnancy, medication/pharmacological studies, cancer, pancreatectomy, and severe inborn metabolic diseases (e.g., PKU, SMA). **Figure 1** shows the two searches and the number of papers remaining after screening and excluding doubles.

### 2.3 Screening and data extraction

**Figure 2:**
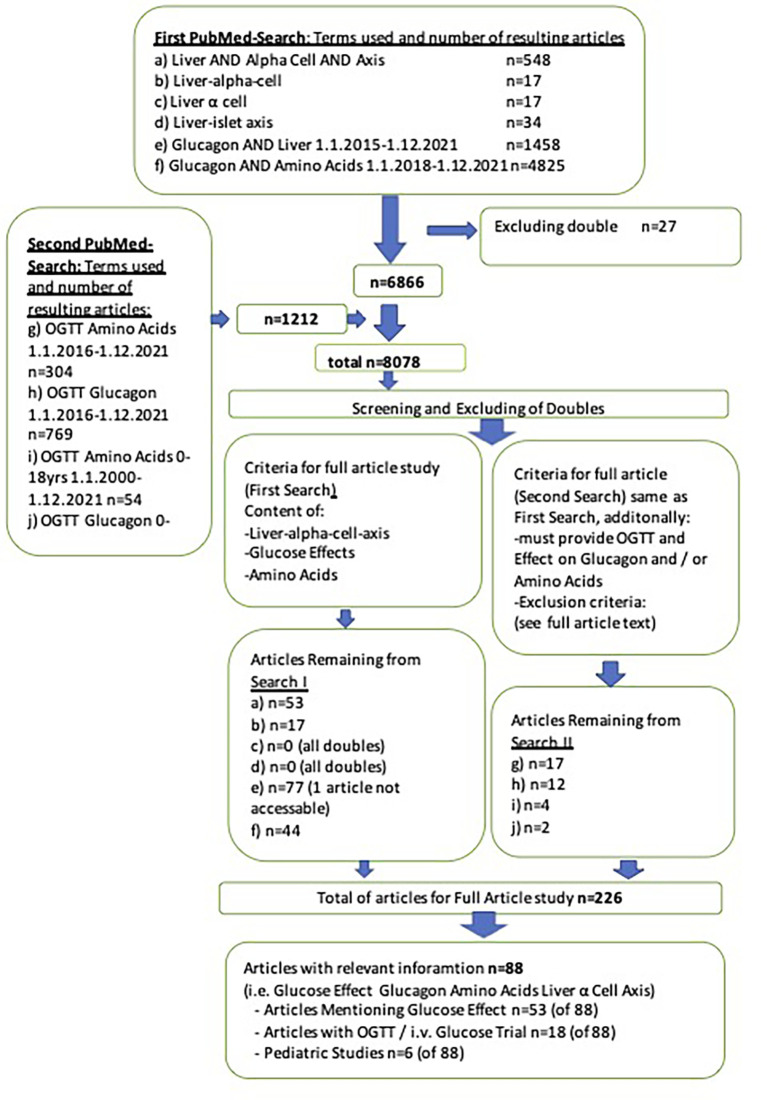
PRISMA-flow chart of literature search.

The two authors in charge of the search screened the first 6866 articles by title and abstract. The title or abstract had to provide information associated with at least one of the following topics: liver-alpha cell axis (LACA), amino acids (AAs), glucagon, or glucose metabolism. One hundred ninety-one articles qualified for the full-text article analysis. After the second analysis, 35 of 1073 articles qualified for full-text analysis and were included in this study. After screening 8078 publications, 88 articles (first search: 67 plus second search: 21) were included (see **Figure 2**). Secondary literature from these articles was studied as well.

### 2.4 Data synthesis

Information on amino acids, glucagon, glucose, and other relevant data, were used to draft the manuscript. As the studies and reviews were significantly heterogeneous concerning populations and methods, we opted for a narrative literature synthesis rather than a meta-analytical approach for interpreting findings.

## 3 Results

### 3.1 Characteristics of literature search

In total 88 articles (first search: 67 plus second search: 21) were included in this review. Secondary literature from these articles was also analyzed and referenced. The literature research *via* PubMed identified 19 articles with standardized glucose administration (i.e., OGTT or intravenous (i.v.) glucose). One article investigated both glucagon and AAs (20). In contrast, 12 other articles examined the effect of glucose administration on glucagon (7, 53–62), and six other articles studied the effect of an OGTT on AAs (63–68). The articles by Knop (7), Junker (54) and Wang et al. (55) were the only trials to include i.v. glucose infusions. Junker (54) mentioned 50g of glucose for OGTT, while the other authors administered 75g glucose OGTT (or 1.75g/kg body weight respectively) (7, 20, 53, 54, 56–68). Six articles included pediatric subjects (22, 62, 68–72). Of these, four reported amino acid levels in children with obesity (22, 68, 69, 72).

### 3.2 Glucose and glucagon under physiological conditions

Under normal conditions, glucagon and insulin counterbalance each other in order to maintain euglycemia (73). A decrease in blood glucose levels simultaneously determines a rise in glucagon levels and a decline in insulin levels (74). Glucagon regulation is complex and controlled by multiple factors (glycemic, paracrine, endocrine, and neural) (39). Hence the results section will initially focus on the isolated glucagon reactions. In hypoglycemia glucagon is secreted from the pancreatic alpha cell (15, 73). Pancreatic alpha cells are well-vascularized islet of Langerhans, which sense glucose concentrations (75). Glucose uptake is mediated by the glucose transporter 1 (GLUT1) in the alpha cell (76, 77). After glucose is taken up by the alpha cell, it is converted to ATP and water in the cell’s mitochondria. Under low blood glucose conditions, ATP concentration drops (18). Alpha cells also contain ATP-sensitive potassium channels (K_ATP_). Variations in intracellular ATP are linked to changes in membrane potential *via* K_ATP_ (78). In low glucose conditions, the K_ATP_ channels in alpha cells are closed or show only mild activation (78). Thus, the opening of voltage-gated Na- and Ca2-channels generate a membrane potential of about ~60 mV. The opening of the Na- and Ca2-channels cause influx of Na and Ca2 and in turn cause the release of glucagon *via* exocytosis and glucagon from glucagon-containing granules (18, 75, 78). The purpose of glucagon secretion during low blood glucose is to increase hepatic glucose production *via* stimulation of glycogenolysis and gluconeogenesis and suppression of glycogenesis and glycolysis (74, 79). The ability to promote the activity of key enzymes responsible for gluconeogenesis (phosphorylase kinase, fructose 1,6-bisphosphatase, glucose 6-phosphatase, phosphoenolpyruvate carboxykinase) has been widely demonstrated (9). Glucagon levels vary along a circadian rhythm. During the physiological morning fasting period, plasma glucagon levels are lower and significant changes in blood glucose levels are required stimuli (80). Hypoglycemia also induces the activity of pancreatic sympathetic innervation and triggers the release of adrenaline from the adrenal medulla. In rodent studies, adrenaline directly stimulates glucagon secretion by binding to alpha1- or beta-adrenergic receptors on the alpha cell (81–84).

The ability of glucagon to elevate blood glucose levels seems to depend on the duration of fasting. The isolated effect of glucagon on gluconeogenesis is short-lasting as it cannot provide substrates (85) and the effect of glucagon subsides after extended fasting periods (22, 86–89). During longer fasting intervals, blood glucose levels mainly depend on cortisol and amino-acids (9, 48). Despite their decreased effectiveness, glucagon levels are elevated in prolonged fasting and starvation (80, 90). The synthesis of new glucogenic AAs from endogenous sources (e.g., muscle, liver) additionally boosts glucagon levels (and induces ureagenesis) (32, 91). In keeping with this, Holst et al. listed the glucose-alanine cycle. Alanine is released from the muscle into the circulatory system during prolonged fasting and then converted into pyruvate in the liver (9, 91). Glucagon regulates alanine transferase (responsible for alanine conversion to pyruvate) and the key enzymes responsible for gluconeogenesis. The cycle, however, depends on the alanine supply rather than glucagon action (9). In periods of starvation, insulin secretion is scarce, and may not inhibit the effects of glucagon (and cortisol) (32).

The suppression of glucagon secretion during hyperglycemia remains only partially understood (92–95). In mixed meals, glucagon levels rise together with blood glucose and insulin levels due to the proteins ingested (80). Conversely, glucagon levels plunge close to zero in healthy individuals following high-carbohydrate diets (8). The previously mentioned cellular mechanism works the other way in hyperglycemia, resulting in high intracellular ATP levels and low levels of ADP in the pancreatic alpha cell. The high ATP causes KATP channels to depolarize the membrane potential, thus inactivating Ca^2+^ and Na^+^-channels. An absent influx of Ca^2+^ and Na^+^ and their lower intracellular concentration hinder glucagon exocytosis and the release of glucagon into the general circulation (78). As glucagon secretion is not only regulated by Ca^2+^ and Na^+^ but also by cAMP, reducing cAMP is presumably involved in inhibiting glucagon secretion (96–99). Yu et al. demonstrated that glucose decreases the cAMP concentration beneath the plasma membrane *via* a direct glycemic effect on pancreatic alpha cells. The mechanism is unclear, but it is reported in mouse and human alpha cells (97). It has been reported that glucagon secretion shows a maximal inhibition at ∼5 mM glucose. When glucose levels exceed this limit, so does glucagon secretion paradoxically (38, 100).

### 3.3 Glucose and glucagon under conditions of metabolic impairment

In individuals with type-2-diabetes-mellitus (T2D), the body’s ability to respond to hypoglycemia appears severely attenuated or completely lost (101, 102). While precise mechanisms remains unclear, paracrine and central pathways have been suggested (103). Normal glucose response in T2D can be restored if glucose control is maintained, as shown by Bolli et al. *via* insulin clamping (pancreatic clamp with somatostatin). This study supports the hypothesis that failure of glucose sensing in T2D is not caused by an intrinsic defect of the alpha cells, but by a lack of metabolic control (104). In T2D, fasting hyperglucagonemia, as well as inadequate suppression of glucagon following carbohydrate ingestion, have been described (44). Hyperglucagonemia is common but not pathognomonic for T2D, as some patients also display normal glucagon level (15). In T2D, glucagon hypersecretion contributes to hyperglycemia (105–109). Therefore, glucagon receptor antagonists have gained attention as potential treatment for T2D (109–112). As with T2D, fasting hyperglucagonemia and increased hepatic glucose production have been observed in other conditions of metabolic impairment (15, 48–50). This includes non-diabetic subjects with obesity, non-diabetic subjects with NAFLD, prediabetic patients, and individuals with metabolic syndrome (15, 45–47, 113). Persistent hyperglycemia on the other hand may directly affect the pancreatic alpha cell *via* intracellular acidification, impaired mitochondrial function, and a resulting dysregulated K_ATP_ channel activity in the alpha cells (114). Of particular note, patients with NAFLD show significantly higher fasting plasma glucagon levels (either normoglycemic or T2D) than normoglycemic and T2D control subjects without NAFLD (113). Also, individuals with hepatic cirrhosis presented with hyperglucagonemia, hyperaminoacidemia, and impaired ureagenesis in response to glucagon (115–117).

### 3.4 Effects of experimental oral & intravenous glucose administration on glucagon

Literature research revealed thirteen articles (see **Table 1**) that investigated the effects of administration of glucose on glucagon (7, 20, 53–62, 71).

**Table 1:** Studies on the dynamics of glucagon after administration of glucose.

Studies on the dynamics of glucagon after administration of glucose										
Authors	Year	Aim of study/article	Sample size	Male/Female	Age (years)	OGTT/ i.v. glucose	(Measured) Metabolites	Timepoints of measurement (minutes)	Results	Authors‘ conclusion
Knop FK (7)	2018	To summarize the physiological regulation of glucagon secretion in humans	n.a	n.a.	n.a.	both	Glucagon, GLP-1, GLP-2, insulin, ‘gastrointestinally mediated glucose disposal’ (GIGD)	n.a.	Glucagon was suppressed normally by i.v. glucose in all groups (independently of glucose tolerance). Glucagon response to oral glucose increases from normal glucose tolerance over impaired glucose tolerance to overt diabetic glucose tolerance. Increased glucagon responses to oral glucose. Hyperglucagonaemia in T2D is aggravated by OGTT, but suppressed similarly as in healthy subjects during IIGI. Elevated fasting plasma glucagon levels contribute to increased basal rate of hepatic glucose production. Demonstration of pivotal role of fasting hyperglucagonaemia in pathogenesis of fasting hyperglycaemia in T2D.	Fasting hyperglucagonaemia is unrelated to the diabetic state, but strongly correlates with obesity, liver fat content and circulating amino acids. Postabsorptive hyperglucagonaemia occurs as a consequence of gut-derived glucagon secretion and/ or glucagonotropic factors. Hyperglucagonaemia seems to occur independently of the diabetic state and rather to be related to obesity- associated disruption of the emerging liver–alpha cell axis (hepatic glucagon resistance) involving amino acids as essential mediators of liver–alpha cell cross-talk (triggering compensatory glucagon secretion from alpha cells). Extrapancreatic glucagon exists.
Wewer-Albrechtsen et al. (20)	2018	Hypothesis was that hepatic insulin resistance (secondary to hepatic steatosis) via defective glucagon signaling/ glucagon resistance would lead to impaired ureagenesis and, hence, increased plasma concentrations of glucagonotropic amino acids and, subsequently, glucagon.	1408	53% males, 47% females	66.2 (mean)	OGTT	AAs (alanine, histidine, tyrosine, glutamine, phenylalanine, isoleucine, leucine, valine) and glucagon, Plasma insulin, ALT, GGT, HOMA-IR, glucagon-alanine-index	0, 30, 120	Plasma concentrations of glucagon and alanine decreased during the OGTT. Absolute concentrations of alanine differed by tertile of HOMA-IR. For middle and upper tertiles of HOMA-IR, fasting plasma concentrations of alanine were higher (0.27 ± 0.06 and 0.28 ± 0.06 mmol/l, respectively), and in these two tertiles alanine concentration decreased in response to glucose intake (change in alanine 120 min after the OGTT: −0.010 [95% CI −0.014, −0.007] and −0.018 mmol/l [95% CI −0.022, −0.015 mmol/l], respectively; p < 0.001 for both). Increasing plasma concentrations of BCAAs associated with increasing plasma concentrations of glucagon.	Plasma concentrations of both glucagon and non- BCCAs (e.g. alanine) during an OGTT were affected by increasing HOMA-IR. Observations support hypothesis that impaired hepatic glucagon signaling, potentially due to fat accumulation in the liver and resulting hepatic insulin resistance. This impairs glucagon’s ability to lower plasma levels of non-BCAAs. Study supports the existence of a liver alpha cell axis in humans: glucagon regulates plasma levels of amino acids, which in turn feedback to regulate the secretion of glucagon. With hepatic insulin resistance, reflecting hepatic steatosis, the feedback cycle is disrupted, leading to hyperaminoacidaemia and hyperglucagonaemia. The glucagon-alanine index is suggested as a relevant marker for hepatic glucagon signaling.
Wagner et al. (53)	2017	To investigate the change in glucagon during oral glucose tolerance tests (OGTTs), hypothesizing that higher postchallenge glucagon levels are observed in subjects with impaired glucose tolerance (in three cohorts of non-diabetic individuals).	Cross-sectional: 4194 total ; Longitudinal study: n=50 (after life-style intervention)	n.a.	n.a.	OGTT	Glucagon	0, 30, 60, 90, 120 (TUEF and TULIP cohort) ; 0 and 120 (MDCS and PPP-Botnia cohort)	66–79% of participants showed suppression of glucagon at 120 min (fold change glucagon120/0 <1) during OGTT, whereas 21–34% presented with increasing glucagon levels (fold change glucagon120/0 ‡1). Participants with non- suppressed glucagon120 had a lower risk of IGT in all cohorts (odds ratio 0.44–0.53, P < 0.01). They were also leaner and more insulin sensitive and had lower liver fat contents.	Lower glucagon suppression after oral glucose administration is associated with a metabolically healthier phenotype.
Junker AE (54)	2015	Summarizes three studies (co-)authored by author. 1) assessed the impact of NAFLD on the incretin effect in patients with or without type 2 diabetes. 2) investigated the influence of cirrhosis on incretin physiology 3) examined the glucagonostatic effect of GLP-1 and its potential glucose- dependency in non-diabetic patients with NAFLD - excluded from this review as glucose-levels were kept at fasting levels.	1) NGT and NAFLD n=10; T2D and NAFLD n=10; T2D only n=8; healthy controls n=10 ; 2) Cirrhosis: n=10; Healthy controls: n=10	1) n.a. ; 2) Cirrhosis: 5/5; Healthy controls: 5/5	1) NGT and NAFLD: 56 (39–63); T2D and NAFLD: 64 (54–65); T2D only: 59 (50–67); healthy controls: 57 (49–60); (mean (span)) ; 2) Cirrhosis: 54±15; Healthy controls: 57±15	1,2) OGTT/ IIGI	1,2)Glucagon, GLP-1, GIP, insulin, plasma glucose, incretin effect	1,2) -15 to +240	1) Fasting glucagon levels were similarly low inpatients with T2D only [4.5 pmol/L(3.0–6.0 pmol/L)] and controls[3.4 pmol/L(1.8–6.0 pmol/L)]. Patients with normal glucose tolerance and NAFLD exhibited immediate glucagon suppression during the first hour of the OGTT and the IIGI [-83 pmol/L (-70to 16 pmol/L) vs.-133 pmol/L(-225 to79 pmol/L)9min,P=0.037]. Controls showed similar immediate glucagon suppression during the first hour of the OGTT and the IGII. Patients with T2D only had delayed and impaired glucagon suppression during the first hour of the OGTT compared to the IIGI[22 pmol/L(-146 to 73 pmol/L) vs. -45 pmol/L (-116 to 27 pmol/L)9min,P=0.027 and 35 pmol/L (-35 to 87 pmol/L)vs.-94 pmol/L (-115 to 43 pmol/L) 9min,P=0.039]. Impaired glucagon suppression was most pronounced in patients with T2D and NAFLD. 2) Patients with cirrhosis and healthy controls had similar incremental changes in glucagon. Plasma glucagon dropped abruptly in both groups following oral as well as intravenous glucose and continued to decrease the following 90 min. A more pronounced suppression of glucagon was seen during IIGI than OGTT.	1) Patients with NAFLD, despite having normal glucose tolerance are characterized by reduced incretin effect, fasting hyperglucagonaemia and impaired handling of ingested glucose. Study emphasizes the role of NAFLD in metabolic dysregulation and suggests an important role for the liver in the regulation of glucagon secretion. 2) In spite of fasting hyperglucagonemia, patients with cirrhosis suppressed glucagon during both OGTT and IIGI, indicating preserved alpha cell sensi-tivity to GLP-1.
Wang et al. (55)	2019	To investigate the progression of obesity-related type 2 diabetes mellitus (T2DM) in rhesus monkeys, especially dynamic changes in insulin and glucagon. Intravenous glucose tolerance test was performed every 6 months to evaluate dynamic changes in glucose, insulin and glucagon levels over 7 years.	52 rhesus monkeys	all males	n.a.	IVGTT	Glucagon, insulin, blood glucose	1, 3, 5, 10, 15, 20, 30, 45, 60	During IVGTTs, glucagon remained consistently elevated in the T2DM group with obesity in all tests, while it increased gradually in the non-T2DM group with obesity and became significantly higher than the lean group in the 7th year test. AUC for glucagon in all tests in the obese T2DM group were significantly higher than the lean and the non-T2DM groups with obesity in year 1, 3 and 5. In the lean group, glucagon immediately decreased after glucose was administered and returned to baseline within an hour. However, in the T2DM group, glucagon decreased after glucose challenge, but then dramatically increased to a level much higher than baseline 1 h later.	Hyperglucagonemia plays an important role in the development of T2D.
Koopman et al. (56)	2019	To examine the association between glucagon responses at baseline and fasting glucose levels 7 years later.	121 (NGT: n=109, IFG: n=12)	50% females	54.1±6.6 at baseline	OGTT	Glucagon (AUCs)	AUCs (0-30 and 30-120)	Glucagon response was differentiated for early (0-30 minutes), late (30-120 minutes) for OGTT. Authors observed an association for the early lack of response and not the late or total glucagon in participants with prediabetes. In NGT Glucagon-level after OGTT steadily declined and reached its lowest level at 60 minutes of OGTT before returning to baseline at around 120 minutes.	Within a population without diabetes, relative lack of glucagon suppression early after a meal was associated with increase of glucose levels over time, suggesting a role of insufficient glucagon suppression in the deterioration of glycemic control.
Engelbrechtsen et al. (57)	2018	To investigate if mutations of the human Ether-á-go-go- related gene (hERG) potassium channel alter glucose homeostasis.	6816 (1329 individuals from the ADDITION-PRO Cohort (NGT: n=708, IFG: n=254, IGT: n=103, IFG and IGT: n=116, T2D: n=148) and 5487 individuals from the Inter 99 cohort)	48% females in ADDITION-PRO cohort and 51% females in Inter 99 cohort	66.3±6.9	OGTT	Glucagon, insulin, blood glucose, GIP, GLP-1	0, 30, 120	The Kv11.1 voltage-gated hERG potassium channel (minor G-allele of rs1805123) was associated with 0.95 pmol/L (− 1.52; − 0.38)) lower fasting glucagon (p = 0.003) and decreased glucagon AUC at 0–30 and 0–120 min (β=−2.73 (−4.73, −0.70), p=0.009 and β=−2.27 (−4.25, − 0.24), p = 0.029, respectively).	Common missense polymorphisms of the Kv11.1 voltage-gated hERG potassium channel (minor G-allele of rs1805123) are associated with alterations in circulating levels of glucagon, suggesting hERG potassium channels play a role in fasting and glucose-stimulated release of glucagon.
Gar et al. (58)	2018	To investigate if glucagon patterns were homogeneous within certain metabolic phenotypes.	285 total (3 metabolic phenotypes: 1) healthy control: n=93; 2) normoglycemic high-risk: n=121; 3) prediabetes/screening-diagnosed type 2 diabetes: n=71	all females	1) 35.3±4.2; 2) 35.2±4.5; 3) 35.9±4.5	OGTT	Glucagon	0, 30, 60, 90, 120	Fasting plasma glucagon was significantly elevated, and early glucagon suppression was diminished in the prediabetes/diabetes group compared with the control group. (Early-suppression glucagon (0–30) (%): 1) 47.6 (32.8–57.9), 2) 41.3 (22.9–58.3), 3) 32.0 (14.5–51.3) - p=0.0055 ; Late-suppression glucagon (30–120) (%): 1) 31.8 (8.9–49.6), 2) 40.9 (14.9–56.7), 3) 47.4 (33.3–63.6) - p=,0.0001.) Late and overall glucagon suppression was smaller in women with isolated IFG compared with both other groups. Total glucagon suppression was similar in all three groups. The five-point glucagon curves in response to oral glucose were heterogeneous between individuals.	Fasting hyperglucagonemia and delayed glucagon-suppression are strongly linked to obesity and metabolic syndrome. Rising glucagon during OGTT may be a rare phenomenon. It occurs in insulin-sensitive individuals with a tendency toward hypoglycemia, but does not necessarily indicate metabolic health.
Kosuda et al. (59)	2022	To investigate glucagon dynamics in patients with postprandial syndrome.	n=14	3 males/11 females	40 (30–49)	OGTT	Glucagon, insulin, blood glucose	0, 30, 60, 90, 120, 180, 240	Plasma glucagon was immediately suppressed by nearly 70% in patients with postprandial syndrome. Authors state that in healthy persons glucagon is reportedly suppressed by approximately 50%.	In patients with postprandial syndrome glucagon suppression is greater than in healthy subjects.
Ichikawa et al. (60)	2019	To clarify the involvement of glucagon in the pathophysiology of DM. OGTT was performed in subjects with NGT, preDM and DM and changes in glucagon and metabolites pre- and post-OGTT were compared.	NGT:n=25, preDM: n=15, DM: n=13	NGT: 12/13 , preDM: 8/7 , DM: 9/4	NGT: 36.±14.69 7 preDM: 54.1±11.5, DM: 60.8±4.8	OGTT	Glucagon, plasma glucose, insulin, glucagon and active GLP-1	0, 30, 60, 90, 120	During OGTT, glucagon levels were less suppressed in DM and preDM than in NGT, whereas no apparent relationship was observed between glucagon and GLP-1 secretion.	Subjects with mild T2D showed elevated fasting glucagon and paradoxical glucagon increase after oral glucose load compared to subjects with normal glucose tolerance.
Jonsson et al. (61)	2021	To examine the impact of gene variants associated with T2D on glucagon-levels during OGTT.	1899 (1346 from ADDITION-Pro cohort, 553 from RigCoh)	709/637 in ADDITION-Pro cohort, 264/289 in RigCoh	66.3±6.9 in ADDITION-Pro cohort, 22.0±2.2 in RigCoh	OGTT	Glucagon	0, 30, 120	*EYA2-variant* was associated with higher 30 min plasma glucagon levels during the OGTT. Authors also identified novel gene locus associated with reduced suppression of early glucagon secretion.	EYA2 locus is associated with increased plasma glucagon levels at 30 min during OGTT, while other variants influence glucagon levels without conferring an increased type 2 diabetes risk.
Manell et al. (62)	2016	Investigation of fasting and postprandial proglucagon derived hormones (e.g. glucagon) in adolescents with obesity along the spectrum of glucose tolerance.	NGT n=23, IGT n=19, T2DM n=4, and age-matched lean adolescents (n=19)	32 males/33 females	10-18	OGTT	Glucagon, GLP-1 and glicentin	-5, 5, 10, 15, 30, 60, 90, 120	Glucagon during OGTT: levels in lean individuals started to decrease between 5 and 10 minutes with suppression close to the observed maximum suppression after 30 minutes. In adolescents with obesity and NGT or IGT, glucagon levels tended to increase during the first 5 minutes of the OGTT. There was no lowering below fasting levels until 30 minutes. In individuals with obesity and T2D, glucagon levels increased during the initial 15 minutes, with no reduction below baseline until 60 minutes.	In adolescents with obesity, glucagon levels are elevated and the progression to T2DM is related to a further increase as well as an early-phase hyperglucagonemic response to OGTT.
Kahn et al. (71)	2021	To determine whether hyperresponsiveness of the beta cell and insulin resistance in youth vs. adults in the Restoring Insulin Secretion (RISE) Study are related to increased glucagon release.	Youth: n=66 Adults: n=350	Youth: 47 (71.2) Adults: 182 (52.0)	Youth: 10-19 (14.2 ± 2.0) Adults: 52.7 ± 9.3	OGTT	Glucagon, glucose, C-peptide, insulin	−10, −5, 10, 20, 30, 60, 90, 120, 150, 180	Mean ± SD fasting glucagon (7.63 ± 3.47 vs. 8.55 ± 4.47 pmol/L; P = 0.063) and steady-state glucagon (2.24 ± 1.46 vs. 2.49 ± 1.96 pmol/L, P = 0.234) were not different in youth and adults. Fasting glucose and glucagon were positively correlated in adults (r = 0.133, P = 0.012) and negatively correlated in youth (r = −0.143, P = 0.251). The absolute glucagon concentrations were lower in youth at multiple time points during the test. This was due to a more rapid decline in glucagon in the first 30 min during OGTT in youth than adults. However, iAUC C-peptide relative to dAUC glucagon across the 3-h OGTT was not significantly different in youth and adults.	Youth with IGT or recently diagnosed T2D (drug naive) have hyperresponsive beta cells and lower insulin sensitivity, but their glucagon concentrations are not increased compared with those in adults. Alpha cell dysfunction does not appear to explain the difference in β-cell function and insulin sensitivity in youth versus adults.

AAs (amino-acids), AAAs (aromatic amino-acids), ALT (alanine transaminase), BCAAs (branched-chain amino-acids), CI (confidence interval), DM (diabetes mellitus), EFAs (essential fatty acids), EYA2 (EYA transcriptional coactivator and phosphatase 2), FFAs (free fatty acids), GGT (gamma-glutamyl transferase), GIGD (gastrointestinally mediated glucose disposal), GIP (Gastric inhibitory polypeptide), GLP-1 (glucogon-like peptide 1), GLP-2 (glucogon-like peptide 2), hERG (human Ether-á-go-go- related gene), HOMA-IR (Homeostatic Model Assessment of Insulin Resistance), IGT (impaired glucose tolerance), IFG (impaired fasting glucose), IIGI (isoglycemic intravenous glucose infusion), IR (insulin resistance), i.v. (intravenous), IVGTT (intravenous glucose tolerance test), OGTT (oral glucose tolerance test), NGT (normal glucose tolerance), T2D (type 2 diabetes mellitus).

Ten articles studying the effect of glucose on glucagon were performed exclusively using an OGTT. Wewer Albrechtsen et al. examined the influence of an OGTT on glucagon and amino acids, demonstrating the relationship between AAs and glucagon in a feedback loop through the liver-alpha cell axis. Elevation of fasting glucagon was associated with lower concentrations of alanine, tyrosine, glutamine, and increased concentrations of BCAAs. Higher tertiles for HOMA-IR were associated with elevated fasting glucagon and a steeper decline in glucagon levels during OGTT than in lower tertiles while maintaining higher levels after 120 minutes (20). In an observational cohort study by Koopman et al. among 121 nondiabetic individuals, glucagon responses at baseline and glucose levels seven years later were investigated (56). During OGTT, glucagon levels dropped in the first 60 minutes, to reach baseline levels again after about 120 minutes (fasting plasma glucagon 9.4 ± 2.9; glucagon iAUC_0-30min_ OGTT −0.23 ± 0.7; glucagon iAUC_30-120min_ OGTT −2.80 ± 3.8; glucagon iAUC_0-120min_ OGTT −2.94 ± 4.5 [all pmol/L, mean ± SD]). Deteriorating glycemic control over time was associated with insufficient glucagon-suppression during OGTT (56, 60) and an early lack of glucagon suppression exists in prediabetes (118). In keeping with this, Ichikawa et al. found glucagon-levels to be less suppressed during OGTT in T2D and prediabetes than in individuals with normal glucose tolerance (NGT) as well as higher fasting glucagon (prediabetes: 34.4 ± 4.6, T2D 44.1 ± 5.0, NGT: 20.6 ± 3.6, all pg/mL). This decrease of glucagon suppression was also reported in two articles investigating genetic variants and glucagon secretion during OGTT (57, 61). Engelbrechtsen et al. explored common variants in the human ether-aí-go-go related (hERG) gene resulting in a dysfunctional Kv11.1 voltage-gated potassium channel. Authors reported the minor G-allele of rs1805123 to be associated with decreased fasting glucagon release: if combined with the minor A- allele of rs36210421 a suppressed glucagon response to increased glucose levels during an OGTT was reported (57). Jonsson et al. performed a genome-wide association study to identify novel loci that affected plasma glucagon levels. The authors documented that higher plasma glucagon levels at 30 min during the OGTT (Beta 0.145, SE 0.038, P = 1.2 × 10–4) were significantly associated with a T2D variant in EYA2, noting a 7.4% increase in plasma glucagon level per effect allele. Jonsson et al. identified a marker in the MARCH1 locus, significantly associated with a reduced glucagon suppression during the first 30 min of the OGTT (Beta − 0.210, SE 0.037, P = 1.9 × 10–8), (8.2% less suppression per effect allele). They also found nine additional independent markers, not previously associated with T2D, that demonstrated suggestive associations with a reduced suppression of glucagon during the first 30 min of the OGTT (P < 1.0 × 10–5) (61).

Wagner et al. investigated the glucagon response in three cohorts (TUEF study, Bonita-PPP study, Tulip study) with a total of 4194 subjects. They found no suppression of glucagon during OGTT (glucagon_120min_) in 21-34% of subjects (in all three cohorts). These individuals were leaner, had a higher insulin sensitivity, a lower risk for impaired glucose tolerance (IGT) (OR 0,44-0,53 in all cohorts, p<=0.009) and lower fasting glucagon levels. The authors concluded that there was an association between non-suppressed glucagon at 120 minutes of OGTT and a metabolically healthier phenotype with lower IGT risk (odds ratio [OR] was 0.44–0.53 in all cohorts, p ≤ 0.009) (53). Comparing glucagon suppression between the groups (i.e., NGT, IFG, and IGT) and in the TUEF study at the intervals 0–30, 0–60, 0–90, and 0–120 minutes, only fold change glucagon120/0 minutes was different. In the PPP- Bonita study, of the 98 individuals with incident diabetes, significantly fewer had increasing or stable glucagon_120min_ than subjects without diabetes (25 vs. 39%, P = 0.002). Glucagon_120min_ in NGT was 15.2 median (12.6-18.9 [IQR/95%CI]), while in IGT it was 13.5 median (11.5-18.1 [IQR/95%CI]). The TULIP study assessed glucagon (fasting and 120 minutes during OGTT) before and nine months after a lifestyle intervention. Lifestyle intervention reduced fasting glucagon (19 vs. 17.5 [median] and 15.2-25.6 vs. 14.4-20.7 [IQR/95%CI]) as well as glucagon after 120 minutes (16.2 vs. 15.2 [median] and 12.4-20.4 vs. 13.4 vs. 18.1 [IQR/95%CI], all pg/mL) (53). Wagner et al. compared their results to those by Faerch et al. (118), that had registered lower early suppression of glucagon (minutes 0-30) and higher late suppression of glucagon (minutes 30-120) in patients with prediabetes and incident diabetes, compared to individuals with NGT (118). The authors found comparable data on late glucagon suppression, however, the TUEF study did not show differences between prediabetes and NGT for shorter intervals (30/0, 60/0, and 90/0). Also, glucagon levels were about two-fold higher than those of Faerch et al. (118). Wagner et al. stated that similar AUCs for glucagon levels and additional controlling for fasting glucagon suggest that fasting glucagon does explain the association between glucagon suppression and insulin sensitivity. Additionally, they found a strong inverse association of hepatic triglyceride content with non-suppressed glucagon_120min_ in their data (51). Similarly, Gar et al. found four patterns of glucagon dynamics that did not match metabolic phenotypes in female patients. While Gar et al. reported fasting hyperglucagonemia and delayed glucagon suppression in prediabetes and T2D (median Q1 to Q3 for fasting plasma glucagon: 6.0 [4.6 to 8.2] vs 7.7 [5.6 to 11.2] in controls); early glucagon suppression: 47.6 [32.8 to 57.9] vs 32.0 in controls [14.5 to 51.3, all pmol/L], respectively. This applied to only 21% of cases and 8% of the control group. Delayed glucagon suppression was clearly associated with obesity and metabolic syndrome. One cluster of seven individuals with low fasting glucagon had rising glucagon levels during the OGTT. The women in this cluster were lean, insulin sensitive, and displayed low plasma glucose (58). The study with the smallest population was done by Kosuda et al., who investigated glucagon response to an OGTT in 17 patients with idiopathic postprandial syndrome (IPS). They reported two types of glucagon dynamics. The first was characterized by lower fasting glucagon and further suppression during OGTT, and the second with fasting hyperglucagonemia and drastic decrease during OGTT. The authors concluded that glucagon suppression in patients with IPS is more substantial than in healthy individuals (59).

Two publications addressed glucagon levels during an OGTT in a pediatric collective, which are presented in the section on pediatric data below (62, 71). Of the studies that included intravenous glucose administration, one study by Wang et al. was on rhesus monkeys. They performed i.v. glucose tolerance tests every six months and measured glucagon levels during tests. In the T2D group with obesity, glucagon remained elevated. In the non-T2D group with obesity, it increased gradually to become significantly higher in the 7^th^ year test. In lean monkeys, glucagon immediately decreased after i.v. glucose and returned to baseline within an hour. In the T2D group, glucagon initially decreased after the glucose challenge to increase to a considerably higher level than baseline after 60 minutes (55). In human studies, Knop reports hyperglucagonemia during OGTT in T2D patients specifying they responded with normal suppression of glucagon during an isoglycemic intravenous glucose infusion (IVGI) (7, 119). The author documents the reproduction of this effect in a number of studies (120–122). Knop found the same reaction in other forms of diabetes (e.g., secondary diabetes after pancreatitis) (7). In a publication on incretin hormones and glucagon in liver disease, Junker included a summary of three separate studies. The first investigated the influence of NAFLD on the previously mentioned hormones in individuals with normal glucose tolerance and or T2D. All patients underwent OGTT (50g glucose) and an isoglycemic intravenous glucose infusion (IVGI). In the first study, only fasting glucagon was measured. Incretin effect in controls was stronger than in NAFLD and T2D. Fasting hyperglucagonemia was associated with NAFLD independent of T2D. Controls and T2D without the liver disease had similar fasting glucagon levels. The second study concentrated on patients with cirrhosis. Fasting hyperglucagonemia was present in cirrhosis and both oral (50g glucose OGTT) and i.v. glucose suppressed plasma glucagon. Junker concluded that cirrhosis complicates the treatment of oral glucose and reduces the incretin effect, possibly contributing to glucose intolerance in cirrhotic patients. The third study included no glucagon measurements (54).

### 3.5 Effects of glucagon on amino acids and vice versa

Glucagon is known to stimulate the influx of AAs into liver cells to provide substrates for gluconeogenesis (106). In order to do so, glucagon stimulates AA transporters expression in the liver for alanine, glutamine, asparagine, and histidine (123, 124). After the influx of AAs into the hepatocytes, the AAs are processed for gluconeogenesis and ureagenesis (85, 125). Under pharmacological blockade of the glucagon receptor signal, plasma concentrations of AAs increase while ureagenesis decreases (13, 28, 126–131). Inhibition of glucagon signaling reduces the expression of genes involved in hepatic AA uptake and AA metabolism, thus resulting in hyperaminoacidemia (10, 13, 14, 17, 28, 132, 133). Wewer Albrechtsen et al., Longuet et al., Kim et al. and Dean et al. reported that amino acids regulate glucagon secretion and the alpha cell mass and its proliferation (10, 14, 16, 17, 22). Most AAs stimulate glucagon and insulin secretion with varying potency (29–33). Additionally, there also seem to be differences among species (34). Otten et al. found fifteen AAs potentially signaling to the alpha cell to increase glucagon secretion. They proposed alanine as the main signal molecule, arguing that, if administered intravenously, it is a potent stimulator of glucagon secretion (134–136). However, in the fasting state, results for alanine remain unclear. While elevated fasting levels of alanine are associated with elevated fasting glucose (137, 138) alanine was not associated with glucagon (136). Elevated glucagon secretion has also been reported following the administration of arginine, cysteine, lysine, glycine, and proline (136). Results for glutamine remain controversial. In cell culture models, it is known to cause alpha cell proliferation but does not cause glucagon secretion in the perfused mouse pancreas (10, 136). Leucine was associated with alpha cell proliferation in isolated mouse islets (10, 17), but the results on glucagon secretion for BCAAs remain inconclusive. BCAAs were reported to not stimulate glucagon secretion (30, 32, 136, 139), except for one study on a perfused rat pancreas (29). In contrast, another study reported significant correlations between postprandial glucagon and leucine, and isoleucine and valine (134). The prolonged administration of AAs led to alpha cell proliferation as shown in isolated pancreatic mouse islets (14), and certain AAs (alanine, glutamine, glutamate, and leucine) were consistently associated with alpha cell proliferation (10, 17). AA concentrations, as shown with BCAA, vary with disease. Patients with NAFLD presented with increased plasma concentrations of BCAAs, aromatic AAs, glutamate, alanine, and lysine, and decreased glycine and threonine (140). Interestingly plasma concentrations of glucagonotropic amino acids were elevated in patients with NAFLD, and AA concentrations correlated with glucagon concentrations (141).

### 3.6 Effects of experimental oral glucose administration on amino acids

Seven articles investigated the dynamics of amino acids during isolated glucose intervention (i.e., OGTT) in the context of LACA (20, 63–68). **Table 2** lists the articles containing information on amino acid dynamics during OGTT as well as their results and conclusions.

**Table 2:** Studies on the dynamics of glucagon after administration of glucose.

Studies on the dynamics of amino acids after administration of glucose											
Authors	Year	Aim of study/article	Sample size	Male/Female	Age (years)	OGTT/i.v. glucose	Amount of glucose administered	(Measured) Metabolites	Timepoints of measurement (minutes)	Results	Authors‘ conclusion
Wewer Albrechtsen et al. (20)	2018	Hypothesis was that hepatic IR via defective glucagon signalling/glucagon resistance would cause impaired ureagenesis and increased plasma concentrations of glucagonotropic amino acids and glucagon.	total: n=1408 (Lower tertile: n=469, Middle tertile: n=470, Upper tertile: n=469)	Lower tertile: 49.7% female, Middle tertile: 50.6% female, Upper tertile: 40.7% female	58.9-74.2 (66.2 mean)	OGTT	75 g	Glucose, glucagon, Four non-BCAAs (alanine, histidine, tyrosine and glutamine), Three BCAAs (isoleucine, leucine and valine), Phenylalanine	0, 30, 120	OGTT did not significantly affect alanine for individuals in the lower tertile of HOMA-IR. For middle and upper tertiles of HOMA-IR, fasting plasma concentrations of alanine were higher (0.27 ± 0.06 and 0.28 ± 0.06 mmol/l, respectively), and in these two tertiles alanine concentration decreased in response to OGTT (change in alanine 120 min after the OGTT: −0.010 [95% CI −0.014, −0.007] and −0.018 mmol/l [95% CI −0.022, −0.015 mmol/l], respectively; p < 0.001 for both).	Higher fasting plasma glucagon concentrations were associated with lower concentrations of certain non-BCAAs including alanine, tyrosine and glutamine, and with higher concentrations of BCAAs.
Sjögren et al. (63)	2021	To identify differences in circulating and skeletal muscle BCAA levels in response to an OGTT in individuals with normal glucose tolerance or T2D.	total n=61 (NGT: n=32; T2D: n=29)	all male	44-69	OGTT	75 g	BCAAs (isoleucine, leucine and valine), Branched-chain α-keto acids (BCKAs),	fasting, 30, 120	Fasting plasma BCAAs were ~10% higher in T2D. Fasting BCKAs did not differ between NGT and T2D. OGTT decreased circulating levels of BCAA and BCKA in NGT but not in T2D.	Disturbances in the BCAA profile are exacerbated by glucose loading. This reveals, that the metabolic inflexibility that characterises T2D encompasses BCAA catabolism
Wang et al. (64)	2019	To characterize the metabolic changes in response to an oral glucose test (OGTT) and assess the associations of these changes with insulin resistance.	total: n=5340	6 subgroups (male: 33-63%)	46.2-47.2	OGTT	75 g	Total of 78 metabolic markers (incl. Amino-acids, Glucose, insulin, lipids, ketone bodies)	0, 30, 60, 120	NGT: almost all measured AAs were decreased during the OGTT, except for alanine. BCAAs (isoleucine, leucine, and valine) and AAA (phenylalanine and tyrosine) showed stronger decrease during OGTT.	Non-diabetic individuals are exposed to a similar adverse postprandial metabolic milieu, as those with T2D
Li et al. (65)	2016	To investigate changes in postprandial AAs and biogenic amine profiles provoked by an OGTT in hyperlidemia patients using targeted metabolomics.	total: n=70	47/33	37.46-57.62	OGTT	75 g	Total of 21 metabolic markers (incl. Alanine, Arginine, Cystein, Glycine, Glutamic acid, Histidine ,Isoleucine, Leucine, Lysine, Methionine, Phenylalanine,Proline, Serine, Tryptophan, Threonine ,Tyrosine, Valine, Asparagine, Creatine)	0, 120	Healthy controls: 4‐hydroxy‐L‐proline, valine, asparagine, tyrosine decreased significantly. Healthy controls: serine, taurine, cysteine and creatine increased significantly. Hyperlidemia: eucine, isoleucine, serine, histidine, lysine, taurine, cysteine and creatine increased significantly. Six metabolites (methionine, dimethylglycine, aminobutyric acid, niacinamide, allantoin and creatinine) can be used as biomarkers for hyperlidemia:	Elevated fasting and postprandial levels of BCAAs during OGTT revealed significant metabolic alterations in the amino acid-metabolism in hyperlipidemia compared to healthy individuals.
Geidestam et al. (66)	2015	Study changes in OGTT-elicited metabolite patterns in obese subjects during a diet induced weight loss study.	total: n=14	3/11	33,5-40,5	OGTT	75 g	All 21 AAs, 11 FFAs	0, 30, 120	Suppression of AAAs is associated with decreased insulinogenic index observed after weight loss (tyrosine: r = 0.72, p = 0.013; phenylalanine: r = 0.63, p = 0.039). Suppression and/or lack of increase in levels of glutamine, isoleucine, leucine and glutamate after OGTT improved towards lean profile. after weight maintenance. Individuals with obesity demonstrated a greater heterogeneity in the OGTT-response before and after weight loss.	Weight loss followed by weight maintenance results in changes of some but not all serum profiles elicitated by OGTT that are different in obese glucose intolerant compared to lean glucose tolerant subjects. Changes coincide with improvements in hepatic or peripheral insulin sensitivity during weight loss and weight maintenance.
Liu et al. (67)	2015	To investigate the metabolic alterations in obesity provoked by an OGTT using targeted metabolomics.	total: n=30	n.a.	18-23	OGTT	n.a.	29 Amino-acids and biogenic amines, 14 FFAs and 14 EFAs	0,30,60,90,120	Compared with the controls, eight amino acids and biogenic amines significantly increased (leucine, valine, isoleucine, phenylalanine, proline, alanine, creatine and asparagine, P < 0.05) in the population with obesity and three metabolites, glutamine, glutamic acid and taurine, decreased (P < 0.05).	Elevated fasting levels and a delayed decrease in AAs during OGTT are important characteristics of metabolic perturbations in obesity. Correlation between postprandial changes in BCAAs with insulin resistance in obesity of importance.
Trico et al. (68)	2017	Identify early metabolic features of insulin resistance in youth and whether they predict deterioration of glycemic control	78 non-diabetic adolescents, 16 in follow-up 2 years later	34/44; 2 year follow up: 9/7	8 to 18	OGTT	75 g	BCAAs (i.e. leucine, isoleucine, valine), lactate, alpha-hydroxybutyrate, beta-hydroxybutyrate	0, 30, 60, 90, 120	In adolescents with reduced insulin sensitivity the decline in BCAAs was blunted throughout the course of the OGTT (p<0.03).	During OGTT α-hydroxybutyrate and BCAA concentrations characterize IR-youth and predict worsening of glycemic control.

AAs (amino-acids), AAAs (aromatic amino-acids), BCAAs (branched-chain amino-acids), CI (confidence interval), DM (diabetes mellitus), EFAs (essential fatty acids), FFAs (free fatty acids), GGT (gamma-glutamyl transferase), HOMA-IR (Homeostatic Model Assessment of Insulin Resistance), IR (insulin resistance), i.v. (intravenous), OGTT (oral glucose tolerance test), NGT (normal glucose tolerance), T2D (type 2 diabetes mellitus).

In a previously mentioned study from 2018, Wewer Albrechtsen et al. analyzed glucagon and eight AAs during an OGTT (20). The study included 1408 adult individuals from the Danish ADDITION-PRO study that underwent a 75g OGTT. Blood samples were drawn at 0, 30 and 120 minutes to analyze serum glucose, glucagon, and eight AAs. The AAs were four non-BCAAs (alanine, histidine, glutamine, and tyrosine), three BCAAs (isoleucine, leucine, and valine), and phenylalanine. During the OGTT, plasma concentration for glucagon and alanine decreased. Alanine concentrations differed by HOMA-IR tertile. Individuals in the lower HOMA-IR tertile were not significantly affected (fasting: 0.26 ± 0.06 mmol/L change in alanine 120 min after the OGTT: −0.003 mmol/L [95%CI −0.007, 0.000 mmol/L], p=0.076). Middle and upper tertiles for HOMA-IR showed higher fasting alanine (0.27 ± 0.06 and 0.28 ± 0.06 mmol/L, respectively). In these tertiles, a significant decrease occurred during OGTT (change in alanine 120 min after the OGTT: −0.010 [95% CI −0.014, −0.007] and −0.018 mmol/L [95% CI −0.022, −0.015 mmol/L], respectively; p<0.001 for both). Authors found statistically significant non-linear associations with fasting glucagon levels for phenylalanine, isoleucine, leucine and valine (p ≤ 0.049). They also found a modifying effect of hepatic insulin resistance on the associations with fasting plasma glucagon (p ≤ 0.040) for alanine, tyrosine, phenylalanine, ‘total non-BCAA’ (alanine, tyrosine, histidine and glutamine), isoleucine, leucine, and total BCAA. Wewer Albrechtsen et al. found that increasing levels of hepatic insulin resistance (but not peripheral IR) (p > 0.166) attenuated the association between glucagon and circulating levels of alanine, glutamine, and tyrosine. This was also significantly associated with hyperaminoacidaemia and hyperglucagonaemia (20). The association between insulin resistance and BCAAs during an OGTT was also investigated by Sjögren et al. They investigated differences in circulating BCAA levels in response to an OGTT between the circulatory system and skeletal muscles (63). For the purpose of this review, the plasma levels of BCAAs were of interest. The study population included thirty-two men with normal glucose tolerance (NGT) and 29 men with T2D. The authors found that the impaired BCAA catabolism in T2D under fasting conditions was exacerbated during OGTT. In NGT, the OGTT resulted in a 37-56% reduction of BCAAs, with no detected changes in patients with T2D. Fasting BCAAs levels (isoleucine, leucine, and valine) were ~10% higher in T2D than in NGT, no significant changes were recorded for corresponding branched-chain α-keto acids (BCKAs). The OGTT decreased circulating levels of BCAA and BCKA in NGT. The authors concluded that a glucose challenge may unmask defects at several steps of BCAA catabolism in T2D. The circulating concentrations of leucine, isoleucine, and derived BCKAs exhibited a positive correlation (r=0.64–0.77) with blood glucose, HOMA-IR, and HbA1c after an OGTT (63). A 2021 study by Wang et al. came to similar conclusions concerning BCAAs. They analyzed 78 metabolic parameters during fasting and during an OGTT. In individuals with NGT almost all measured AAs decreased during the OGTT, except for alanine. BCAAs (isoleucine, leucine, and valine) and aromatic amino acids (AAA) (phenylalanine and tyrosine) showed a more noticeable decrease (15 to 45%) than the other amino acids (not specified by authors) (6 to 10%) at 120 minutes. In insulin resistance (IR), the BCAAs were higher at baseline and had a weaker decrease at 2 hours. Individuals with NGT and IR showed a less favorable metabolic profile than insulin-sensitive individuals with NGT (P<0.0006 and consistent when stratified by sex). Individuals with diabetes or prediabetes showed marginal differences in metabolic responses concerning branched-chain amino acids. Authors concluded, subjects with NGT and IR have a similar metabolic pattern and cardiovascular risk level as in T2D. The article did not include absolute values of AAs (64).

Two studies focused on AA dynamics during OGTT in the context of obesity. Liu et al. measured AAs after an OGTT in a cohort of 15 young adults with obesity (18-23 years) and compared values with those from 15 lean controls. In the group with obesity, baseline (i.e., fasting) BCAAs (leucine, valine, isoleucine) as well as phenylalanine, proline, alanine, creatine, and asparagine were significantly increased (P<0.05), while glutamine, glutamic acid, and taurine were decreased (P<0.05). During the OGTT 2h-glucose was positively associated with leucine (r=0,84, P<0.05) and tryptophan (r=0.77, P<0.05) in controls. In the group with obesity, changes in arginine and histidine were positively associated with parameters for obesity (P<0.05). Increasing fasting glucose was positively associated with changes in histidine concentrations (P=0.004). HOMA-IR correlated with changes in leucine, isoleucine, phenylalanine, lysine, and histidine during the OGTT (67). The second study, by Geidenstam et al. analyzed 21 AAs during an OGTT in 14 individuals with obesity during weight loss. Results suggested different metabolic patterns during weight loss and weight maintenance, and only a few profile changes towards the lean reference. They reported that after weight-loss a suppression of aromatic amino acids was associated with decreased insulinogenic index (tyrosine: r = 0.72, p = 0.013; phenylalanine: r=0.63, p=0.039). Moreover, OGTT-elicited suppression or lack of increase in levels of glutamine, isoleucine, leucine, and glutamate improved towards the lean profile in weight maintenance following weight loss, and improved glucose tolerance (AUC Glutamine (weight-loss: r=0.76, p=0.003 and weight-maintenance: r=0.85, p=0.0002, respectively). Subjects with obesity’s response to OGTT before and after weight loss was more heterogeneous than in lean patients, although reduced during weight maintenance (66). One study was included individuals with known hyperlipidemia and compared their AA dynamics during OGTT to healthy controls. Li et al. investigated 21 metabolic parameters before and after an OGTT (plasma samples at minutes 0 and 120). In healthy controls, the levels of 4‐hydroxy‐L‐proline, valine, asparagine, tyrosine decreased, and the concentrations of four amino acids (serine, taurine, cysteine and creatine) increased significantly after the OGTT. In HLP there were significant increases in leucine, isoleucine, serine, histidine, lysine, taurine, cysteine, and creatine, while reductions in six metabolites (methionine, dimethylglycine, aminobutyric acid, niacinamide, allantoin, and creatinine) were noted after the OGTT. Li et al. reported that their data positively associated the postprandial changes in isoleucine and HOMA-IR. The study only listed fasting values and used graphics for measurements at 120 minutes and their association to clinical parameters (e.g., insulin concentrations) (65). The study on the effect of an OGTT on BCAAs in a pediatric population by Trico et al. is listed in section 3.8. on pediatric data (68).

### 3.7 Further studies on experimental glucose administration

In their study on the role of glucagon and muscle wasting in critical illness, Thiessen et al. infused critically ill patients with insulin and glucose, failing to lower glucagon, instead raising it with parenteral administration of amino acids. The authors suggested the effect of adrenaline and cortisol to avoid a decline in glucagon levels after glucose administration. They concluded that during critical illness hyperglucagonemia increases hepatic AA-catabolism without affecting muscle wasting or blood glucose (142). A study by Kelly et al. included two mathematical models of glucagon effectiveness and sensitivity from an OGTT (not included in the previous sections. These models were used to calculate how various degrees of patient glucagon-sensitivity and effectiveness might affect serum glucose and glucagon concentrations during IVGTT and insulin infusion tests. These suggested that the models could provide a mathematical platform from which the effect of glucagon during a glucose test may be predicted (143). In another publication, Lund et al. examined the glucagon of ten pancreatectomy patients and ten healthy controls. They found glucagon present in patients without a pancreas, demonstrating the existence of extrapancreatic glucagon. The authors suggested that this gut-derived extrapancreatic glucagon may play an unrecognized role in diabetes secondary to total pancreatectomy (144).

### 3.8 Pediatric data

Four studies focused on amino acids in a pediatric population. The study by Suzuki et al. included 26 children with obesity (15 male, age 122.2 ± 4.2 months; 11 female, age 122.9 ± 4.1 months). Elevated branched-chain AAs (BCAAs) leucine, isoleucine, and valine are associated with impaired glucose tolerance and hyperuricemia at early stages of pediatric obesity (72). In this study, HOMA-IR positively correlated with BCAAs, phenylalanine, tryptophan, methionine, threonine, lysine, alanine, tyrosine, glutamate, proline, arginine, and ornithine. In children with obesity and decreased HOMA-IR, levels of BCAAs, aspartic acid, alanine, tyrosine, glutamate, and proline decreased, but levels of glycine and serine increased after six months of lifestyle intervention (i.e., nutrition and exercise, no medication). After intervention in children with obesity and high HOMA-IR, all AA-levels declined (72). Cosentino et al. reported that leucine (19% (p=0.015)), isoleucine (21% (p=0.024)), and valine (21% [p=0.025]) as well as aromatic AAs (AAA, i.e., phenylalanine and tyrosine) were more elevated in children with obesity compared to healthy controls. A lifestyle-intervention program only showed negligible differences in BCAA- and AAA-changes (p>0.05) (145). Goffredo et al. found that plasmatic BCAAs negatively correlated with peripheral and hepatic insulin sensitivity. Dysregulation of BCAAs in adolescents with obesity was considered a characteristic of NAFLD and, therefore, a predictor of an increase in liver fat content (69). The only study to report changes in amino acid levels in a pediatric population during an OGTT was by Trico et al. (see **Table 2**). The study reports BCAA levels and included 78 non-diabetic children and adolescents aged 8-18 who underwent an OGTT (1,75g/kg body weight, up to 75g). Sixteen participants underwent a second OGTT two years later. Insulin sensitivity was estimated from the OGTT using Whole-body insulin sensitivity index (WBISI). In adolescents with a lower WBISI, fasting BCAA levels had increased. During the OGTT, BCAAs showed a more blunted decline after glucose administration in individuals with a lower WBISI (WBISI effect p<0.03 for all BCAAs). BCAA curves were not significantly different between insulin-sensitive and insulin-resistant individuals during the OGTT (146).

We found two publications on glucagon dynamics during an OGTT in a pediatric population. The study by Manell et al. investigated the plasma levels of glucagon, GLP-1, and glicentin in adolescents with obesity and T2D. In line with adult data, the authors found that adolescents with T2D and obesity had fasting hyperglucagonemia twice the level of the NGT group. Adolescents with obesity and NGT had 30% higher fasting glucagon than controls, glucagon levels increased with a decline in glucose tolerance. Fasting glucagon did not differ between NGT and IGT. During the OGTT, glucagon levels in lean adolescents decreased between minutes 5 to 10. Maximum suppression was achieved after 30 minutes. In adolescents with obesity (both NGT and IGT), glucagon levels showed an increasing trend in the first five minutes without declining below baseline in the first 30 minutes. In adolescents with obesity and T2D there was an increase in the first 15 minutes and no decline below baseline until 60 minutes after the start of the OGTT. Boxplots and fasting glucose concentrations are indicated for each subpopulation (62). The second study’s objective was to determine how the hyperresponsiveness of the beta cell and the insulin resistance in youth compared to adults (both with IGT or T2D) was due to an increased glucagon release. Data was gathered from the RISE study (66 youth and 350 adults), and patients on antidiabetic drugs were excluded. The younger population was 10-19 years of age with a Tanner stage >1. Fasting glucagon and steady-state glucagon did not differ between younger and adult participants. While fasting glucose and glucagon were positively correlated in adults (r=0.133, p=0.012), they negatively correlated in the younger group (r= -0.143, p=0.251). At comparable fasting glucagon levels, the 10-19 years olds had higher C-peptide levels and a lower insulin sensitivity. During a hyperglycemic clamp (blood glucose at around 11.1. mmol/L and >25 mmol/L), glucagon suppression was similar between the pediatric and the adult collectives. In the course of the OGTT, glucagon decreased in both groups, although absolute glucagon levels were lower in the young patients at multiple time points. Notably, the decline was steeper in the younger population in the first 30 minutes, but AUC for glucagon did not differ significantly between the two groups. Following an arginine infusion, glucagon levels in this study increased in youth and adults, but the response was significantly lower in the pediatric population. Higher fasting glucagon concentrations were associated with lower insulin sensitivity for both age groups. The authors had hypothesized that hyperglucagonemia would significantly contribute to the hyperresponsiveness of beta cells and insulin resistance in youth. As glucagon concentrations were lower in youth, the authors dismissed their hypothesis of direct beta cell stimulation *via* glucagon, suggesting a greater beta cell sensitivity towards glucagon in youths (71).

## 4 Discussion

The LACA is an established concept and disruption of the LACA may trigger hyperglucagonemia and hyperaminoacidemia. To this day, the underlying mechanisms are incompletely understood (22). As amino acids are considered to be drivers of the LACA (i.e., they stimulate glucagon secretion and alpha cell proliferation), their dynamics during glucose administration is of interest (29–33).

In healthy adults without steatosis and with NGT, fasting amino acid levels are normal and AA levels decrease readily during OGTT. However, while results for specific AAs are partly inconclusive (e.g., alanine) (22, 64, 140), BCAAs have consistently been linked to metabolic impairment (e.g., HOMA-IR as surrogate for hepatic insulin resistance) (147). Fasting BCAA-levels are elevated in adults with obesity, NAFLD, and T2D and are also associated with cardiovascular diseases (148). In individuals with diabetes and prediabetes, BCAA catabolism is impaired and plasmatic levels decrease slower during OGTT as in individuals with normal glucose metabolism (20, 64, 67). Hence, the OGTT might be useful in demasking defects in BCAA-catabolism (e.g. T2D) (63). BCAA catabolism involves a reversible and an irreversible process. The first takes place either in the cytosol or the mitochondria and is catalyzed *via* a branched-chain aminotransferase (BCAT). The second process is catalyzed in the mitochondria by branched-chain keto-acid-dehydrogenase (BCKDH), leading to formation of substrates that enter the Krebs cycle (149–151). In individuals with diabetes, BCAT and BCKDH expression may be decreased due to genetic variants (152, 153). However, a deranged function of the two enzymes has also been related to increased levels of insulin, fatty acids, and proinflammatory mediators, linking these hallmarks of obesity-associated metabolic impairment to BCAA catabolism and the LACA (154).

While a number of publications on fasting glucagon exist, there is very limited data on its dynamics after glucose administration. In general, glucagon levels are expected to decline when blood glucose levels rise. In adults, this response has been constantly observed after i.v. glucose administration, while responses to oral glucose vary. Of note, T2D is not only attributed to beta-cell dysfunction but also alpha-cell dysfunction. In keeping with this, hyperglucagonemia was repeatedly reported during OGTT in T2D and has been attributed to an incretin effect as well as to intestinal glucagon secretion (7, 119). Additionally, endogenous glucose production is higher during OGTT than after i.v. glucose administration (7). The lack of early glucagon suppression, especially within the first 30-60 minutes, may be an early and reliable hallmark of prediabetes and IGT in adults (56, 60, 118), although contradictory results question this (53). It is of interest that increased glucagon levels upon oral glucose challenge can also be found in healthy individuals and has even been associated with a lower risk for impaired glucose tolerance (IGT) (53). A potential explanation may be a protective effect of glucagon against acute hypoglycemia to counterbalance a strong insulin response to the glucose load and genetic variants involved in ion-channels regulating glucagon secretion (55, 56, 58, 155). Still, higher fasting glucagon and late glucagon-suppression during OGTT are consistent findings in prediabetes, T2D, obesity and the metabolic syndrome (53). The degree of obesity has been positively associated with glucagon levels, independent of insulin-resistance (25) and individuals suffering from hepatic dysfunctions display fasting hyperglucagonemia without altered glucose tolerance (156). As NAFLD has been demonstrated to be even more strongly associated with hyperglucagonemia than T2D, Wewer-Albrechtsen et al. considered the different degrees of liver fat in patients with T2D the reason for varying glucagon levels. However, the extent to which the steatotic liver is also partially resistant to the hyperglycemic actions of glucagon is unknown (22). As hyperglucagonemia is associated with fasting hyperglycemia and elevated HbA1c-levels in adults (22), the question arises if hyperglucagonemia precedes the impairment in glucose metabolism. In adults, the glucagon receptor seems to remain functional for the glucose pathway while the AA-pathway is impaired (157). Glucagon effects are mainly exerted *via* glycogenolysis *via* molecular pathways that are clearly separated from those of gluconeogenesis and ureagenesis (22). In *in vitro* studies, glucagon modulated beta cell function as well as endocrine hormonal function and parasympathetic levels (158).

Of note, dysregulated glucagon secretion is not as significant in adolescents as in adults with T2D. This speaks against a causal role of alpha cell dysfunction in the gradual progression from normal to impaired glucose metabolism and eventually T2D (159). Moreover, contrary to adults, glucagon was negatively associated with fasting glucose in a study by Stinson et al., as they investigated the influence of childhood obesity in 4012 Danish individuals (age 6-10 years). While fasting glucagon was associated with cardiometabolic risk markers (e.g., BMI SDS, body fat percentage, liver fat percentage, triglycerides, blood pressure), it was not associated with hyperglycemia. Thus, hyperglucagonemia might precede impairments in glucose regulation (160). This finding is in line with the pediatric data from the RISE study (71). Kahn et al. also reported an inverse relationship between glucagon and fasting glucose in their collective of 10-19 year old patients. In the RISE study there was no difference in glucagon suppression during OGTT between the age groups, however, arginine administration resulted in significantly lower glucagon levels in the pediatric population. In youth with IGT or newly diagnosed T2D, hypersecretion of beta cells and reduced insulin sensitivity have been described. At comparable glucagon levels as adults have, they also display higher C-peptide levels. Kahn et al. concluded that alpha cell dysfunction in youth does not explain beta cell dysfunction but suggests a greater glucagon-sensitivity in the pediatric population (71). In a pediatric population with obesity and T2D, glucagon-levels showed an early rise during OGTT (62). Manell et al. concluded that insulin or glucose were not responsible for hyperglucagonemia, but gut-derived glucagon or an altered glucagonotropic response to GIP. Additionally, a blunted GLP-1 response to affect glucagon was ruled out, as neither the response of GLP-1, nor insulin correlated with the glucagon-response. In this study, the lower GLP-1 response in adolescents with obesity, independent of glucose tolerance, corroborates previous findings from adult studies (48, 161). Pediatric studies only reported fasting concentrations of AA levels (22, 68, 69, 72). In accordance with adult data, elevated fasting BCAAs correlated with obesity and insulin resistance (22, 72) and were predictors of liver fat content (69). This indicates similarities of BCAAs in the role of metabolic diseases between pediatric and adult patients.

The limitations of this study were a relative paucity of studies on glucose and the LACA, the small number of pediatric studies, and the type of study design (i.e., mainly cross-sectional). Only one article by Dean included a single specific paragraph on the effect of glucose on the LACA (36). Most studies focused on fasting levels of glucagon and/or AAs. One challenge in studies involving the measurement of glucagon is quantifying exact plasma levels. Measurements have conventionally been performed with radioimmunoassay (RIA), deemed unreliable, especially at lower concentrations (51, 52, 60). Wagner at al. reported glucagon levels two times higher than Faerch et al. (118), both studies were performed in 2016 using different assays (53). The enzyme-linked immunosorbent assay (ELISA) may produce more accurate results (60). The main findings of this systematic review include a synopsis on what is known on the relationship of glucose and the liver-alpha cell axis. Certain aspects (e.g., inverse association between glucagon and glucose in youth vs. adults, change of AA-levels with age) hint towards potential differences between adults and children concerning LACA and pathophysiology and need further investigation (69, 71, 162, 163). Many aspects of glucagon-secretion (e.g., in hyperglycemia) are still incompletely understood (92–95). While hyperglycemia might directly attenuate alpha cell function, the consequences for the LACA remain unclear (114). Impaired glucagon response during OGTT is not only present in patients with T2D, but also in healthy individuals (58). The results on AAs in the fasting state remain partly inconclusive, but BCAAs are associated with metabolic impairments and show different dynamics in an altered LACA (63). In conclusion, the concept of the LACA is of great interest but still understudied. The LACA may provide better insight into metabolic diseases, and the dynamics of glucagon and amino acids during standardized glucose challenge tests may hold a predictive or diagnostic value.

## Data availability statement

The original contributions presented in the study are included in the article/supplementary material. Further inquiries can be directed to the corresponding author.

## Author contributions

Conceptualization, TP, DF and DW; Methodology, DW, TP, AL and NS; Analysis, TP, DF, KG and AS; Writing—Original Draft Preparation, TP, DF, VJ and DT; Writing—Review and Editing, TP, KG, KMö, KMa, HM, EA, CDand NS; Supervision, DW; Project Administration, TP and DF. All authors discussed the results and commented on the manuscript. All authors have read and agreed to the published version of the manuscript.
